# Transient Changes in Bacterioplankton Communities Induced by the Submarine Volcanic Eruption of El Hierro (Canary Islands)

**DOI:** 10.1371/journal.pone.0118136

**Published:** 2015-02-11

**Authors:** Isabel Ferrera, Javier Arístegui, José M. González, María F. Montero, Eugenio Fraile-Nuez, Josep M. Gasol

**Affiliations:** 1 Departament de Biologia Marina i Oceanografia, Institut de Ciències del Mar, CSIC, Barcelona, Spain; 2 Instituto de Oceanografía y Cambio Global, Universidad de Las Palmas de Gran Canaria, Las Palmas, Spain; 3 Department of Microbiology, University of La Laguna, La Laguna, Spain; 4 Instituto Español de Oceanografía, Centro Oceanográfico de Canarias, Santa Cruz de Tenerife, Spain; Universidade Federal do Rio de Janeiro, BRAZIL

## Abstract

The submarine volcanic eruption occurring near El Hierro (Canary Islands) in October 2011 provided a unique opportunity to determine the effects of such events on the microbial populations of the surrounding waters. The birth of a new underwater volcano produced a large plume of vent material detectable from space that led to abrupt changes in the physical-chemical properties of the water column. We combined flow cytometry and 454-pyrosequencing of 16S rRNA gene amplicons (V1–V3 regions for Bacteria and V3–V5 for Archaea) to monitor the area around the volcano through the eruptive and post-eruptive phases (November 2011 to April 2012). Flow cytometric analyses revealed higher abundance and relative activity (expressed as a percentage of high-nucleic acid content cells) of heterotrophic prokaryotes during the eruptive process as compared to post-eruptive stages. Changes observed in populations detectable by flow cytometry were more evident at depths closer to the volcano (~70–200 m), coinciding also with oxygen depletion. Alpha-diversity analyses revealed that species richness (Chao1 index) decreased during the eruptive phase; however, no dramatic changes in community composition were observed. The most abundant taxa during the eruptive phase were similar to those in the post-eruptive stages and to those typically prevalent in oceanic bacterioplankton communities (i.e. the alphaproteobacterial SAR11 group, the Flavobacteriia class of the Bacteroidetes and certain groups of Gammaproteobacteria). Yet, although at low abundance, we also detected the presence of taxa not typically found in bacterioplankton communities such as the Epsilonproteobacteria and members of the candidate division ZB3, particularly during the eruptive stage. These groups are often associated with deep-sea hydrothermal vents or sulfur-rich springs. Both cytometric and sequence analyses showed that once the eruption ceased, evidences of the volcano-induced changes were no longer observed.

## Introduction

Submarine volcanic activity results in the release of dissolved and particulate substances, as well as heat into the ocean that can be discharged either continuously (chronic plumes) or occasionally (event plumes) [1] potentially leading to abrupt changes in the physical-chemical properties of seawater and strongly affecting the marine biota. Microorganisms are recognized to play key roles in such environments, yet few studies have characterized the microbial communities that inhabit geologically active marine environments, partly because of the difficulty associated with sample collection. While most of the knowledge on the biogeochemistry of underwater volcanic activity comes from the study of highly evolved deep-sea hydrothermal vents continuously releasing high-temperature reduced hydrothermal fluids (“black smokers”) [2–10], there is little information on event plumes because direct observations of submarine eruptions are rare since they are difficult to predict and monitor and usually occur in remote locations.

El Hierro, the youngest of the Canary Islands, is located in the Northeastern Atlantic Ocean above the presumed location of the Canary Island hot spot, a mantle plume that feeds upwelling magma just under the surface. Seismic and volcanic activity has been continuously documented since 1990 when geophysical monitoring of the island started [11]. In summer 2011, El Hierro began an intense episode of seismic activity that caused more than 12000 earthquakes. As a result, an eruption took place in October 2011 and gave rise to a new shallow submarine volcano of ca. 650 m located 1.8 km south of the island [12]. Initial geophysical surveys of the volcanic eruption were followed by a series of hydrographic cruises, which allowed the study of the changes in the seawater’s physical and chemical parameters as well as their effects on the marine ecosystem. The discharge of high temperature hydrothermal fluids, magmatic gases and volcanic particles during October and November produced warming of the water column and dramatic changes in seawater chemistry, including a significant decrease in pH and oxygen and an increase in iron and nutrients near the volcano [12–13]. These physical-chemical anomalies had strong effects on some pelagic communities. Dead fish were observed floating on surface waters, no fish schools were acoustically detected within the affected area [12] and the diel vertical migration of zooplankton was disrupted [14]. Furthermore, preliminary results reported that the activity of the local microbial communities was also significantly altered. Small picophytoplankton, i.e., *Prochlorococcus* and *Synechococcus*, showed a significant decline in abundance at depths > 75m compared to far-field unaffected stations [12]. Conversely, heterotrophic prokaryotes seemed to increase with depth at stations affected by the volcanic emissions [12]. In order to further characterize the effects of the eruption on the bacterioplankton communities, we combined flow cytometry and 454-pyrosequencing of 16S rRNA gene amplicons to monitor the area around the volcano through the eruptive and post-eruptive phases (November 2011 to April 2012). The effects of this disturbance on prokaryote abundance, activity, diversity and community structure are presented here.

## Materials and Methods

### Sampling

**Fig 1:**
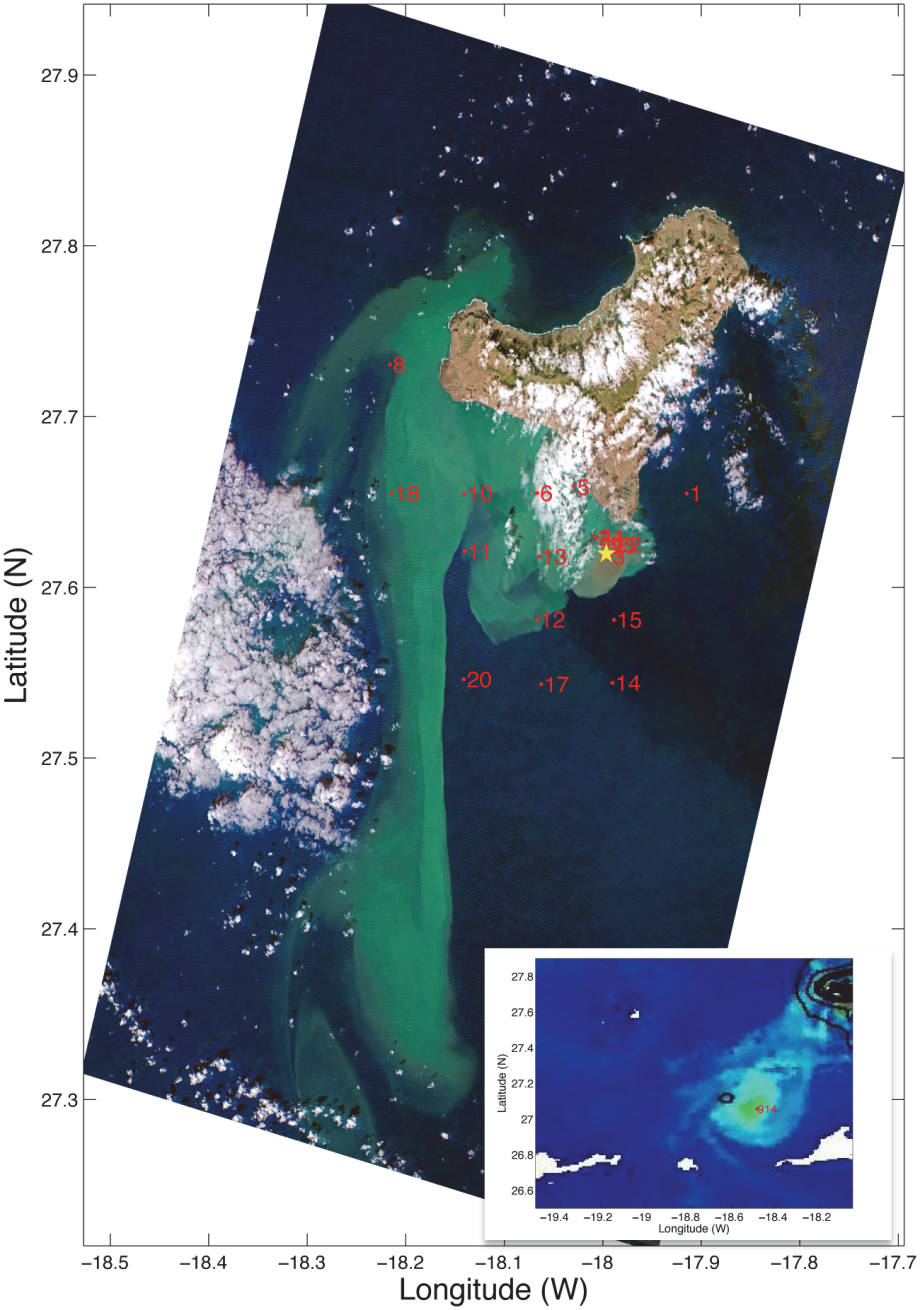
RAPIDEYE© color composite image acquired on October 26, 2011 showing the island of El Hierro. The location of the volcano (yellow star) and of the stations sampled during leg BBC3 (red dots) is indicated. The inset map shows chlorophyll concentration on November 06, 2011 and was acquired by NASA Terra MODIS and processed by the Marinemet project.

The samples were collected in seven oceanographic surveys starting three weeks after the onset of the eruption until the complete cessation of the volcanic unrest (Bimbache (BBC) cruises BBC3, 4–9 Nov 2011; BBC5, 16–20 Nov 2011; BBC8, 13–15 Jan 2012; BBC10, 9–12 Feb 2012; BBC12, 24–26 Feb 2012; and Guayota (GYT) cruises GYT2, 17 Mar 2012; GYT3, 28 Apr 2012). Cruises were carried out by the Spanish Institute of Oceanography with the authorization of the Spanish Government. BBC cruises were performed from aboard *R/V Ramon Margalef* whereas the GYT cruises were performed from aboard the *R/V Atlantic Explorer*. The environmental variables measured include temperature, oxygen, salinity and transmittance and have been published elsewhere [12–14]. Based on satellite and CTD profile data, samples were collected in stations located in the area most affected by the plume and in a control station located in a less affected area east of the island (Station 1) (Fig. 1). Temperature and dissolved oxygen depth profiles along the Bimbache cruises in Station 3 (above volcano) and Station 1 (control) are presented in S1 Fig. Samples for flow cytometric determination of prokaryote abundance were collected in all cruises. Samples (1.6 ml) were preserved with paraformaldehyde (2% final concentration), left 10 min in the dark to fix, deep frozen in liquid nitrogen and stored at—80°C. For DNA analyses, samples could only be collected during cruises BBC3, BBC10, BBC12 and GYT3. About 10 l of seawater were sequentially filtered through a 3-μm pore-size polycarbonate filter (Poretics) and a 0.2-μm Sterivex filter (Millipore) using a peristaltic pump. The Sterivex units were flash frozen in liquid nitrogen and kept at—80°C until extraction was performed.

### Flow-cytometric analyses

Once in the lab, fixed samples were thawed, stained with Syto13 (Molecular Probes) in the dark for a few minutes, and run through a BD FACSCalibur cytometer with a laser emitting at 488 nm. High and Low Nucleic Acid content prokaryotes (HNA, LNA) were identified in bivariate scatter plots of side scatter (SSC-H) versus green fluorescence (FL1-H). Picocyanobacteria were discriminated in plots of orange fluorescence (FL2) versus red fluorescence (FL3) and were subtracted from HNA prokaryote counts. For statistical analyses, the data were grouped depending on three factors: time of sampling, sample location and depth. Multifactor analysis of variance for abundance of prokaryotes and HNA cells with these three factors with Tukey-Kramer *post hoc* comparison at the 5% significance level was performed in R (http://www.R-project.org). Data was also plotted using R.

### Nucleic acid extraction and sequencing

The Sterivex units (Millipore) were filled with 1.8 ml of lysis buffer (50 mM Tris-HCl pH 8.3, 40 mM EDTA pH 8.0, 0.75 M sucrose) treated with lysozyme, proteinase K and sodium dodecyl sulfate. Nucleic acids were extracted with phenol and concentrated in an Amicon 100 (Millipore) as described in Massana et al. [15]. DNA was quantified spectrophotometrically (Nanodrop, Thermo Scientific) and a subsample was used for pyrosequencing at the Research and Testing Laboratory (Lubbock, TX, USA; http://www.medicalbiofilm.org) using the bTEFAP method by 454 GL FLX technology as described previously [16]. Primers 28F (5’-GAGTTTGATCNTGGCTCAG-3’) and 519R (5’- GTNTTACNGCGGCKGCTG-3’) generated amplicons spanning the V1 to V3 regions of the bacterial 16S rRNA gene (∼500 bp), and primers 341F (5′-GYGCASCAGKCGMGAAW-3′) and 958R (5′-GGACTACVSGGGTATCTAAT-3′) were used to amplify archaeal fragments spanning the V3 to V5 regions (∼600 bp). The generated pyrosequencing data were processed using the QIIME (Quantitative Insights Into Microbial Ecology) pipeline [17] as described in Sánchez et al. [18]. After an ID was assigned to each sample using a bar code, a sequence filtration step was performed before denoising. Sequences were removed from the subsequent analyses if they were shorter than 150 bp, had an average quality score < 25 calculated in sliding windows of 50 bp, or had an uncorrectable barcode or > 3 ambiguous bases. The remaining sequences were run through Denoiser to reduce the impact of pyrosequencing errors [19]. Curated sequences were then grouped into operational taxonomic units (OTUs) or phylotypes using UCLUST [20] with a minimum identity of 97%. A representative sequence from each phylotype was chosen by selecting the most abundant sequence in each cluster. The resulting representative sequences were checked for chimeras using ChimeraSlayer [21] in mothur [22]. The identity of 16S rRNA phylotypes was determined using the RDP Classifier [23] implemented in QIIME. BLAST was also used for certain unclassified OTUs as some lineages were not correctly classified by RDP. OTUs represented by one single tag (singletons) were discarded to avoid potential artifacts in diversity estimates. Likewise, OTUs assigned to chloroplasts or mitochondria were removed. Venn Diagram Plotter (http://omics.pnl.gov/software/VennDiagramPlotter.php) was used to generate area-proportional Venn Diagrams. Chao1 diversity metrics and rarefaction curves were computed in QIIME and plotted in Kaleidagraph (v.4.1). Non-metric multidimensional scaling (nMDS) plots were performed and plotted in R (Vegan package) [24]. Phylogenetic trees were constructed with RAxML [25] using the GTR substitution matrix (implemented as GTRGAMMA) and an alignment made with MUSCLE [26] that was previously trimmed using the Gblocks software [27] to eliminate highly diverged regions. Sequence data has been deposited in the MG-RAST public database (http://metagenomics.anl.gov/) under ID numbers 4600697–4600744 (Project Name: Hierro submarine volcano).

## Results and Discussion

### Background information

The submarine eruption off the island of El Hierro started on October 10^th^, 2011. Geophysical surveys determined that on October 23^rd^ the active volcano was located at a depth of 350 m at 27°37’07”N—17°59’28”W. In January 2012, the cone had risen to a depth of 130 m and in February it reached its maximum elevation of 88 m below sea level [28]. In order to oversee the effects of the eruption on the surrounding waters, physical-chemical data and biological samples were collected from the volcanic unrest until the eruption had ceased. The first hydrographic cruise took place three weeks after the eruption (Leg BBC3) when the strongest bubbling episode occurred. Additional samples were collected in late November (BBC5), January (BBC8), February (BBC10, BBC12) March (GYT2) and April (GYT3).

Immediately after the eruption, changes in sea surface reflectance (SSR) due to the discharge of hydrothermal fluids, magmatic gases and volcanic particles, were observed by satellite [13–14,29]. Despite some activity being recorded through March 5^th^, the waters along the south bay of the island were significantly cleaner in early February [14]. Furthermore, based on physical-chemical profiles [13–14] and the measured microbiological parameters (see below), we observed that by January the situation seemed significantly restored. Thus, and from here on, we refer to samples collected in November (BBB3 and BBC5) as the eruptive phase and to samples collected from January to April (BBC8, BBC10, BBC12, GYT2 and GYT3) as the post-eruptive phase.

During the eruptive phase, scientists observed warming of the water column and dramatic changes in seawater chemistry, including a significant decrease in pH and oxygen and an increase in iron that was more pronounced towards the southwest of the island [13]. The CTD profiles revealed a strong thermocline around 80–90 m depth and a clear deoxygenation from ~75 to ~175 m that was particularly pronounced in stations near the volcano [12] (S1 Fig.). Reduced species of sulfur, iron and manganese from volcanic fluid are oxidized quickly when mixing with seawater, which results in oxygen depletion as well as acidification [13]. The thermocline weakened in the post-eruptive phase coinciding with the winter period as typically occurs in the region as a result of surface cooling [30]. No anomalies in oxygen profiles were observed in the post-eruptive stages. Overall, physical-chemical data indicates that about 2 months after the eruption the oceanographic conditions returned to normal.

### Effects on abundance and activity of bacterioplankton

**Fig 2:**
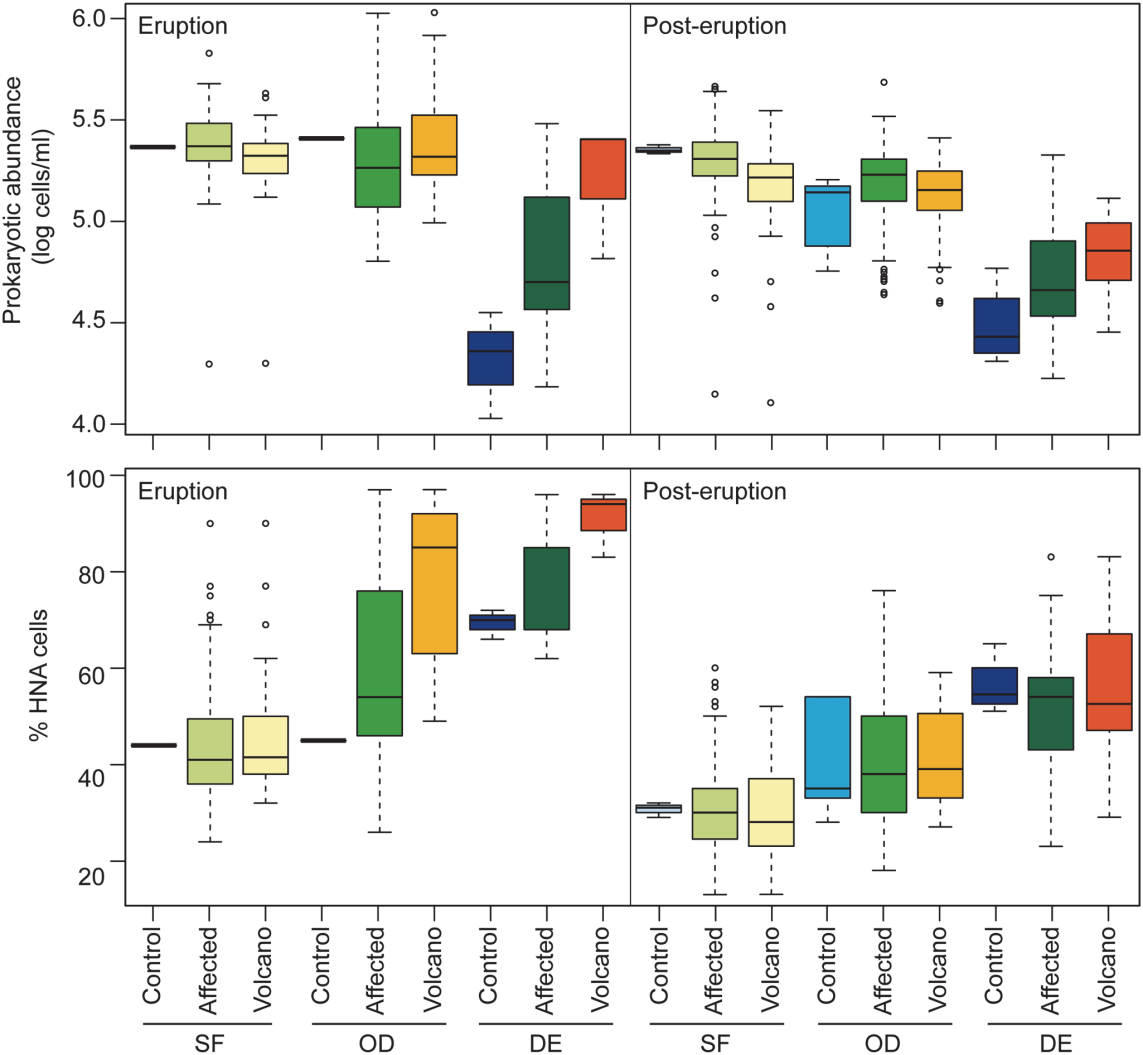
Distribution of prokaryotic abundace (cells ml^-1^ in log units) and the percentage of high-nucleic acid content cells (% HNA) in samples collected during the eruptive phase (left pannels) and the post-eruptive phase (right pannels). The samples are grouped in different categories by depth (SF: subsurface, 0–70 m; OD: oxygen depleted waters, 70–200 m; DE: deep waters, 200–1900) and location (Control: stations in the control zone, Affected: stations in all affected areas, Volcano: affected stations in the vicinity of the volcano; see S1 Table).

The effects of the eruption on the abundance of bacterioplankton in the surrounding waters were monitored by flow cytometry in samples collected from the volcanic eruption until it had ceased. Furthermore, we measured nucleic acid content as a single cell-based proxy of cell activity. Previous investigations have shown that the cells with a high nucleic acid (HNA) content tend to be more active cells [31–32]. Yet, they also represent versatile bacteria with larger and more flexible genomes [33]. A total of 536 samples were analyzed including stations in the affected zone and waters outside the main influence of the eruption (e.g. Station 1) (Fig. 1, S1 Table). Samples were collected at different depths from surface to bathypelagic waters. Based on physical-chemical (temperature, salinity, density, oxygen) and biological (bacterial abundance) parameters the samples have been grouped into three depth categories: subsurface waters (0–70 m), oxygen depleted waters (70–200 m) and deep waters (200–1900 m). Analysis of variance including three factors revealed that there were significant differences (p<0.001) in the abundance of heterotrophic prokaryotes between sampling periods (eruption and post-eruption), sampled area (control, affected and volcano) and between depths (subsurface, oxygen depleted and deep waters). In general, the number of prokaryotes was higher during the eruption than in the post-eruption stages (Fig. 2). Differences were more evident when comparing only the control station with stations in the vicinity of the volcano (Stations 3, 4, 21, 22, 23, 24) (Fig. 2). Oxygen depleted and deep waters were in general more affected than subsurface waters. Likewise, significant differences were found in the percentage of presumably more active cells (HNA cells) between sampling periods and between depths, but no significant differences were found between areas. Nevertheless, the percentage of HNA cells was higher during the eruptive phase in the affected zone and near the volcano, particularly at medium depths and deep waters (Fig. 2). Tukey-Kramer *post hoc* comparisons indeed revealed differences in the fraction of HNA cells between the control and the volcano stations. Despite Station 1 being sampled as a control station, a certain influence of the eruption was observed in the oxygen profiles (S1 Fig.). Additionally, Ariza et al. [14] found by analyzing satellite reflectance that the control zone was affected by small turbidity pulses during the strongest eruptive episodes. Yet, Station 1, placed outside the main influence of the eruption, is considered to be the control zone for reference.

**Fig 3:**
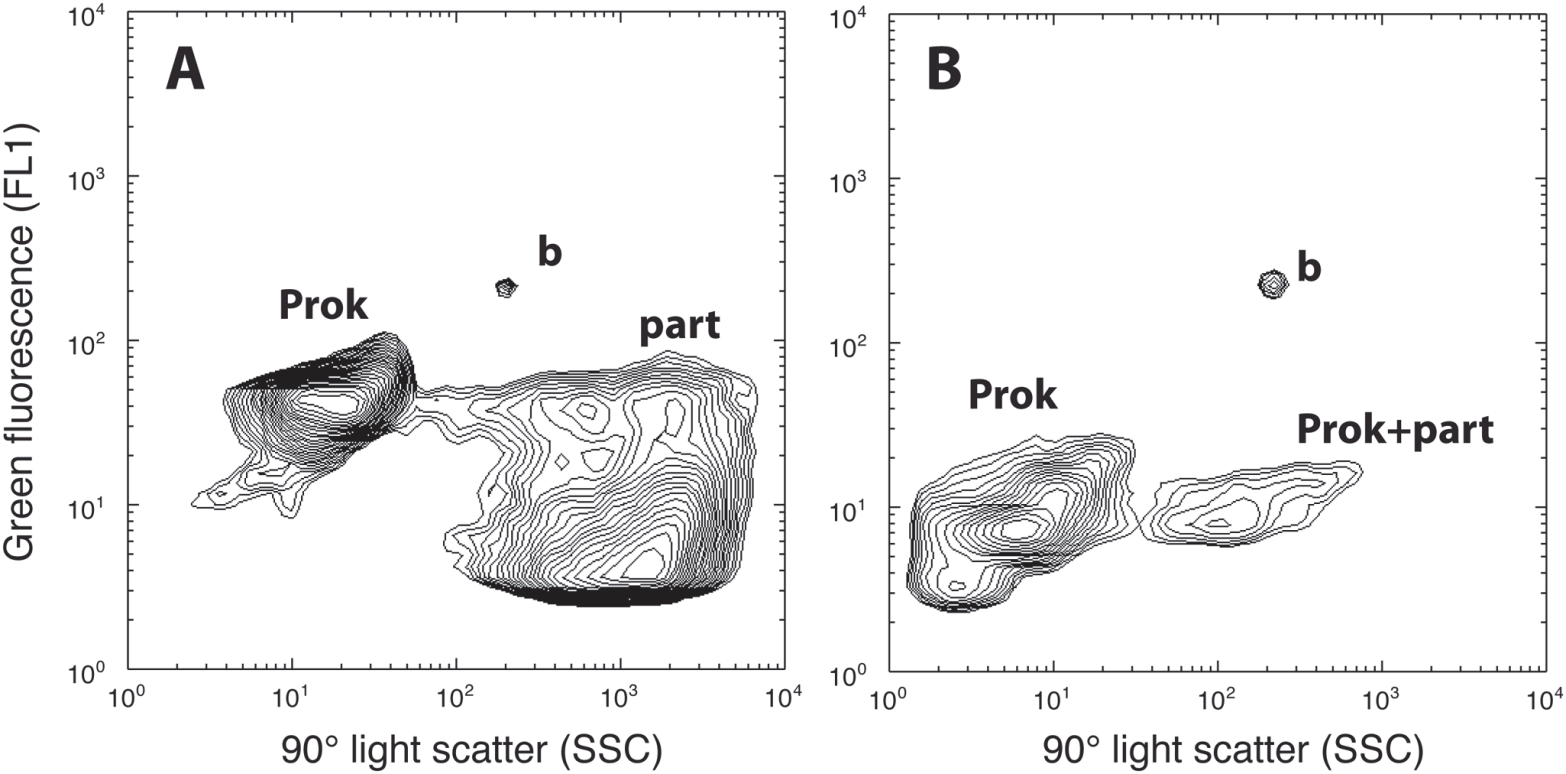
Two examples of Syto13-stained bacterioplankton samples (A: cruise BBC3–St.3, 0 m; B: cruise BBC3–St.17, 70 m) as seen by the flow cytometer in plots of nucleic-acid-based green fluorescence (FL1) versus particle side scatter (a surrogate of particle size). The typical prokaryotic signals (Prok) and the reference beads (b) are accompanied by likely inorganic vent-derived particles (part) and HNA cells likely attached to particles (Prok + part).

Flow cytometric analyses also revealed the presence of two types of particles that were distinct from the HNA or LNA prokaryotic populations typically observed in bacterioplankton cytograms. One is characterized by particles with high SSC and relatively low fluorescence, likely representing inorganic particles (Fig. 3A). This population was observed associated with the discharge of vent material and appeared mostly in stations closer to the volcano (Stations 3, 4, 21, 22, 23, 24) as compared to the rest of stations (p < 0.001). Another distinct population appeared with high SSC and relatively high FL1 (Fig. 3B) that could represent cells attached to these particles. The particles would confer high scatter signal to the prokaryotes, and these were detected in significantly higher amounts in the volcano zone (p <0.0001). Fig. 3 also illustrates the difference in relative nucleic acid content of the whole of the prokaryotes nearest the volcano (Fig. 3A) as compared to elsewhere (Fig. 3B).

The eruption likely promoted an increase in the abundance and activity of heterotrophic prokaryotes during the eruptive phase, notably in depths closer to the volcano (70–200 m) and in deeper waters (200–1900 m). The values returned to normal levels in the post-eruptive period. Santana-Casiano et al. [13] reported a striking enrichment of Fe(II) and nutrients at stations over the volcano during the eruptive phase that could explain the higher values in prokaryotic abundance and activity observed. We do not have data from before the volcanic eruption to compare, but Baltar et al. [34] had reported values of around 24–46% of HNA in epi- and mesopelagic waters of the subtropical northeast Atlantic Ocean near the Canary Islands. These values are within the range of values observed in the post-eruptive stages. Differences between eruption and post-eruption periods could also be attributed partly to seasonality. However, the fact that the HNA values were significantly higher near the volcano supports the hypothesis that the observed changes in the percentage of HNA cells were to a large extent a consequence of the eruption. In fact, extraordinarily high mean HNA values were observed in these waters during the eruptive phase in depths closer to the volcano and deeper waters (79% and 91%, respectively), and these values were significantly different from those of the control zone (Fig. 2).

### Effects on diversity of bacterioplankton

Twenty-four samples collected during 4 of the 7 hydrographic cruises were selected for pyrosequencing. Samples correspond to different stations and depths within the epipelagic (0–200 m) layer in the vicinity of the volcano when it was active (Leg BBC3) and in the following months when its activity had decreased (Legs BBC10, BBC12, and GYT2). Samples were also obtained in the far-field Station 1 in legs BBC10 and BBC12. After a rigorous quality control (see Material and Methods), a total of 213994 bacterial (average 8916 per sample, range 2502–22216) and 80610 archaeal (average per sample 3359, range 365–8761) 16S rRNA high-quality tags were kept and analyzed. Pyrosequencing of all bacterial and most archaeal amplicons was successful, but unfortunately two archaeal samples (BBC3_St.3_0m, BBC10_St.1_800m) resulted in a low number of reads. Clustering of reads into OTUs resulted in a total of 2521 different observed bacterial OTUs ranging between 285 and 1191 per sample (average 572). Overall, bacterial diversity was greater than archaeal diversity. For Archaea, a total of 566 OTUs were distinguished with an average of 158 per sample (range 36–362) but the number of archaeal reads was also lower than for Bacteria. The OTU diversity estimate is a function of the sampling effort and, in fact, we did find a correlation between the observed richness and the sequencing depth (R^2^ = 0.69, p = 0.03). For that reason, we normalized each dataset for comparative purposes.

**Fig 4:**
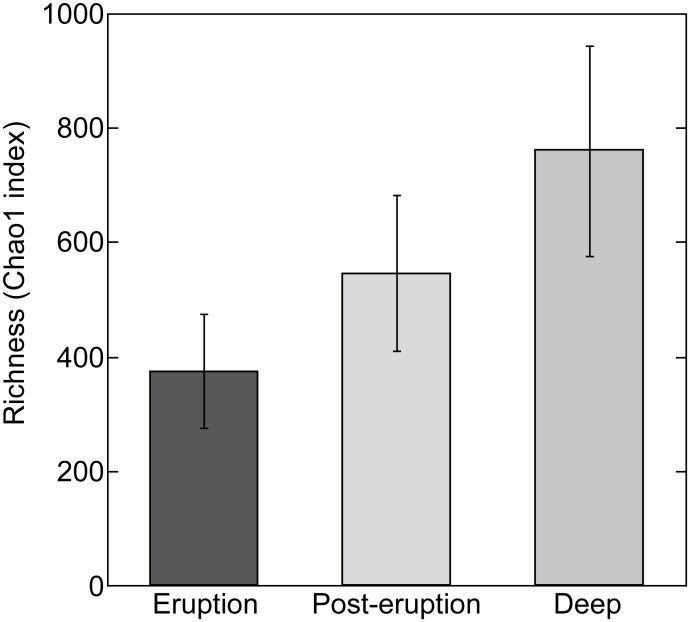
Bacteria richness estimates (Chao1) by type of samples: epipelagic samples from eruption (eruption), epipelagic samples from post-eruption (post-eruption) and mesopelagic samples (deep).

When alpha-diversity was computed at the minimum sequencing depth for the Bacteria dataset (2500), we observed that samples collected during the eruption contained overall less bacterial richness than samples collected in the following months. Mesopelagic (800 m) samples collected in the control station contained higher bacterial richness than epipelagic (0–200 m) samples of the same stations (Fig. 4). Archaeal richness in eruption and post-eruption samples was within the same range (average 181 and 178 respectively) but much more variability was observed in this dataset (S2 Fig.). Contrarily to Bacteria, Archaea in the far-field deep station were less rich than in epipelagic samples collected at the same time. However, only one deep sample could be included in the comparison (samples that resulted in a low number of reads were excluded from this comparison). Rarefaction curves were asymptotic indicating that we retrieved most of the diversity present (S3 Fig.) but this trend may have been influenced by the removal of all singletons. A large proportion of diversity in the environment corresponds to the low-abundant organisms of the rare biosphere [35] often appearing as singletons. By removing them we might have underestimated diversity, but as a tradeoff we reduced the potential artificial inflation of diversity estimates by spurious OTUs associated with pyrosequencing errors [36]. Yet, we must point out that we did not perform ultra-deep sequencing since the main goal of our study was to determine whether the volcanic eruption led to changes in diversity, rather than accurately describing the rare biosphere. However, we cannot rule out that we might have underestimated the effect of the eruption on diversity by the limited depth of sequencing.

### Effects on bacterioplankton community structure

**Fig 5:**
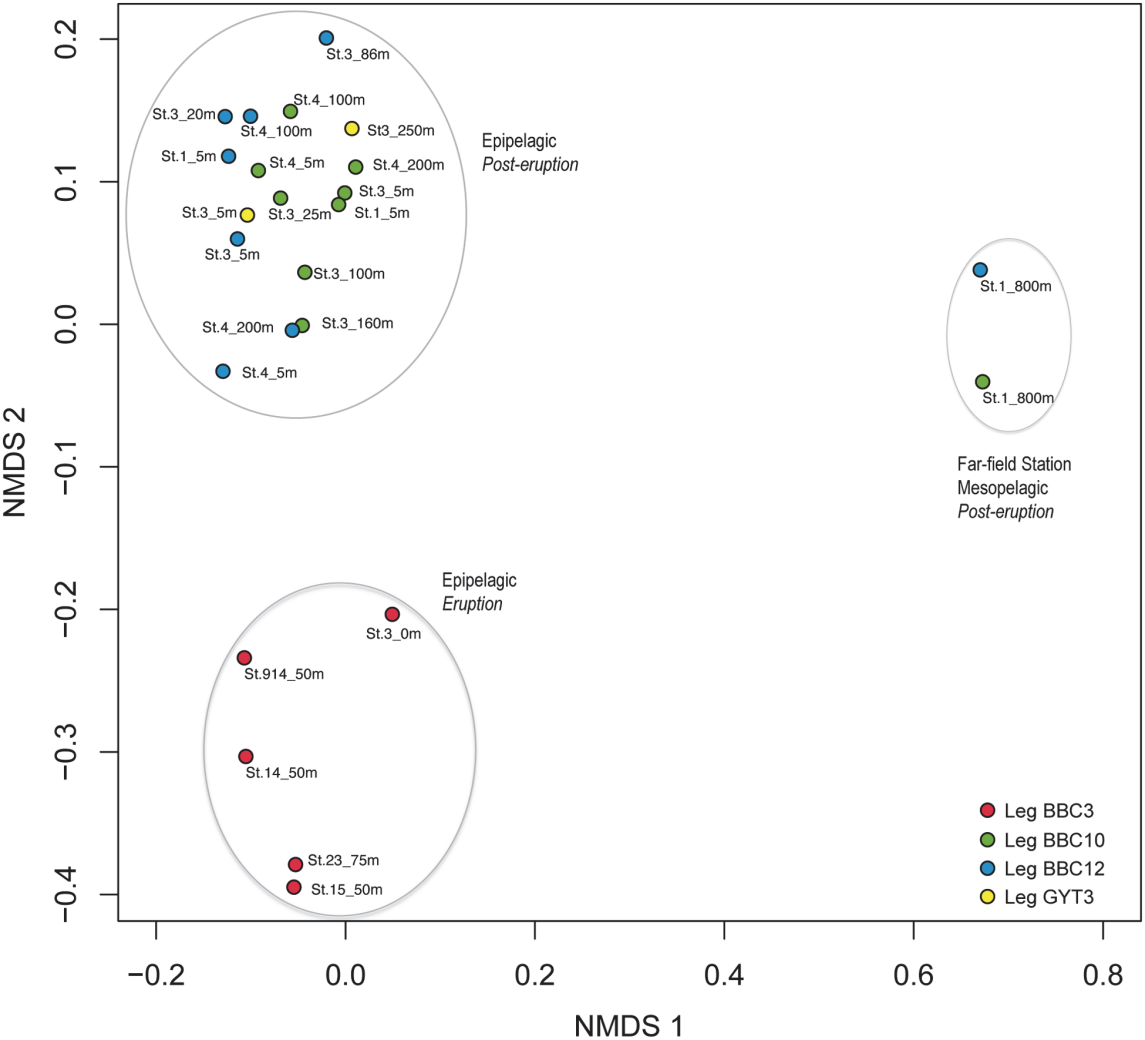
Non-metrical multidimensional (nMDS) plot based on the OTU distributions of the bacterial dataset. The position of samples reflects how different bacterial assemblages are from each other based on their distance in a two-dimensional plot. Distance is derived from the Bray-Curtis similarity coefficients calculated from the square root transformed relative abundance of each OTU. Samples are indicated by station number and depth. Color code indicates the cruise.

**Fig 6:**
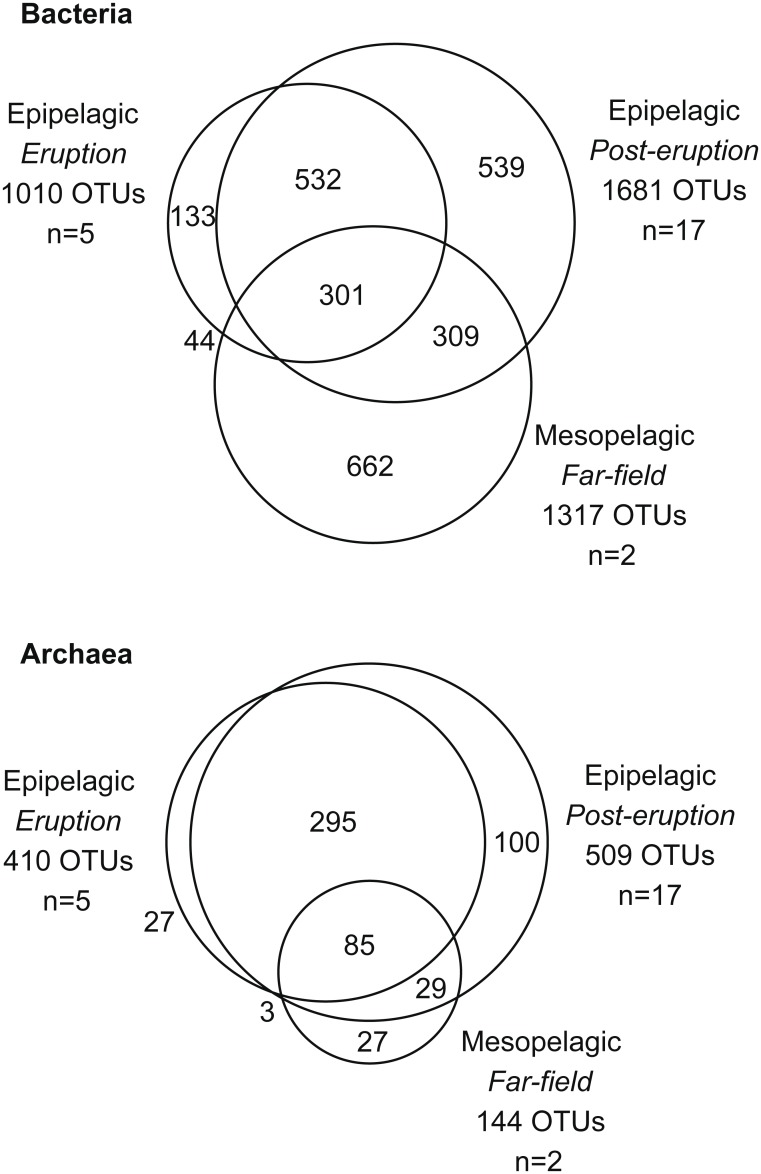
Venn diagrams of shared 16S rRNA gene based OTUs between the three groups of samples: epipelagic during eruption, epipelagic during post-eruption and mesopelagic during post-eruption for Bacteria (top) and Archaea (bottom). The number of OTUs and of samples (n) in each group is indicated.

Differences in microbial composition (beta-diversity) were assessed using OTU-based metrics. Bray-Curtis dissimilarity matrices (bacterial and archaeal) were constructed based on the square root transformed relative abundance of each OTU. The distance between samples was visualized using non-metric multidimensional scaling (nMDS). Visualization of the bacterial Bray-Curtis dissimilarity matrix revealed the presence of three distinct groups of samples according to sampling time and depth (Fig. 5). The first group included all the epipelagic samples from the eruption time, the second group included all epipelagic samples collected in post-eruption cruises, including the far-field sample from Station 1, and the third group clustered the two mesopelagic samples (800 m) collected as reference. Unfortunately, samples from the less affected area during the eruptive phase are not available and thus we cannot discount the possibility that the grouping of samples might partially be influenced by seasonality or other factors as well [37]. However, the presence of certain groups typically associated with hydrothermal systems supports the hypothesis that, as for abundance and activity, the volcano induced some changes in bacterial community structure. For Archaea, deep ocean samples were different from subsurface samples but no clear clustering between eruptive and post-eruptive periods was observed (S4 Fig.). The lower number of sequences analyzed for Archaea could explain this observed lack of effect, yet previous studies have shown than around 1000 sequences per sample and between 30–300 OTUs are sufficient to accurately reveal patterns of beta-diversity in aquatic systems [38–39]. We had sequence numbers below these values only for two of the archaeal samples and nearly identical results were obtained when these were removed from the analyses (data not shown). Therefore, we can conclude that geochemical changes in seawater did result in changes in bacterial community structure but no effect was observed for archaeal communities. Venn diagrams confirmed that a higher proportion of OTUs were shared between samples for Archaea than for Bacteria (Fig. 6). Our data also suggest that, as for abundance and activity, bacterioplankton communities in the area surrounding the volcano were restored in terms of community structure shortly after the eruption. In addition, although the eruption resulted in changes in bacterial communities, the induced differences were less dramatic than, for example, the natural differences found between subsurface and deep waters (Fig. 5).

### Effects on bacterioplankton community composition

While beta-diversity analyses allow depicting differences in community structure, assigning an identity to each OTU provides insights on how these communities differ taxonomically. Taking into account the whole Bacteria dataset, we found that most sequences were related to the phyla Proteobacteria (68.4%), Cyanobacteria (15.8%) and Bacteroidetes (5.9%). Within the Proteobacteria, the most abundant classes were the Alpha- (54.8%) and Gammaproteobacteria (10.0%), but the Beta- (0.1%), Delta- (2.6%), Epsilon- (0.3%) and Zetaproteobacteria (<0.1%) were also present. Overall the most abundant group was the Rickettsiales (SAR11 group, Alphaproteobacteria) making up 52.0% of reads with a total of 611 different OTUs out of the 2521 total OTUs. The Bacteroidetes were largely represented by members of the Flavobacteriia (70% of total Bacteroidetes) and less by the Sphingobacteria (10%). Other groups present with abundances >1% were the Actinobacteria (2.1%) and uncultured group SAR406 (2%). Chloroflexi, Planctomycetes, Gemmatimonadetes, Lentisphaerae, Nitrospirae and SAR202 were also detected at low abundances (<1%).

**Fig 7:**
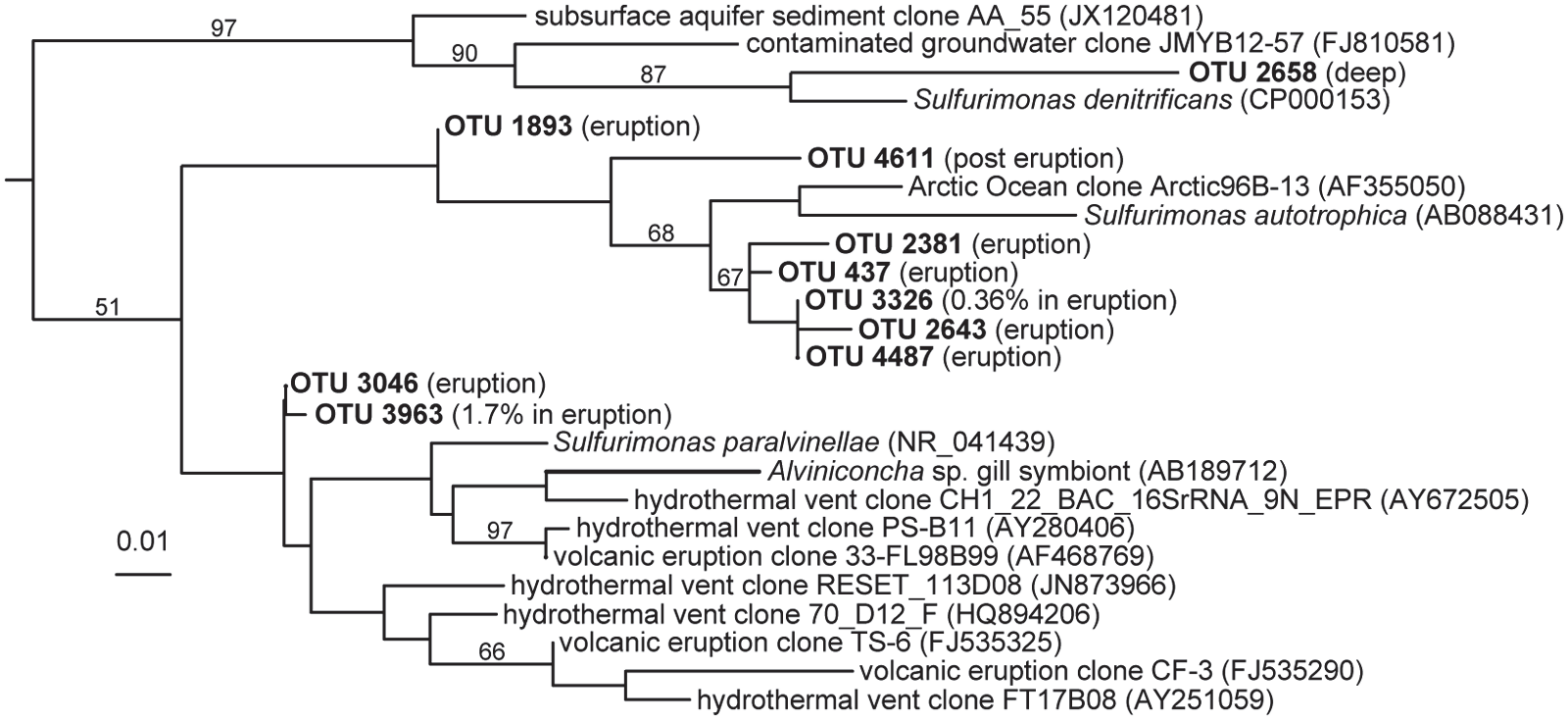
Maximum-likelihood tree of Epsilonprotebacteria 16S rRNA gene sequences retrieved from the bacterial dataset. Each sequence from this study (labeled as OTU number) is representative of clustered sequences at 97% cutoff. The group of samples (eruption, post-eruption or deep samples) where it was detected is shown in parentheses. Reference sequences from GenBank database are indicated by their accession number in parenthesis. The sequence of *Wolinella succinogenes* served as outgroup (GenBank accession number M88159) and is not shown. The scale bar indicates substitutions per site.

Despite the majority of samples being dominated by Proteobacteria, interesting differences were observed at lower phylogenetic levels and when grouping samples by sampling period (S2 Table). The percentage of Alphaproteobacteria was significantly lower in samples collected in November (average 39%) than in samples collected months later (average 56%) (ANOVA, p<0.05). Among these, the relative abundance of the SAR11 group was also lower. Likewise, the relative abundance of Bacteroidetes decreased on average two-fold in samples collected during the eruption compared to months afterwards. The Epsilonproteobacteria made up almost 2% of bacterioplankton communities in November but were hardly present in either epi- or mesopelagic samples collected months after the eruption. Interestingly, the few OTUs found in the post-eruption period were different than those present during the eruption (see Fig. 7). Members of this proteobacterial class are not commonly found in marine planktonic communities but typically dominate deep-sea hydrothermal environments and are known to play significant roles in carbon and sulfur cycling in such ecosystems [40]. Likewise, members of the candidate division ZB3 often associated to oxygen minimum zones and sulfidic environments [41–42] were also detected in the water column at very low abundances. Sequences related to the Zetaproteobacteria (iron-oxidizers associated with seamounts) [43] were detected in the water column at very low abundances in the post-eruption period. Compounds released during the eruption included inorganic sulfur, hydrogen, reduced iron, manganese and ammonium, all of which can serve as a source of energy for some of these organisms, which were likely accompanying the volcanic emissions and thrived in the water column during and after the eruption.

Two phyla, the Thaumarchaeota (58.5%) and Euryarchaeota (41.5%) dominated the Archaeal dataset. Among the Thaumarchaeota, 94% of reads where closely related to the genus *Nitrosopumilus*. Most Euryarchaeota OTUs were classified within the class Thermoplasmata, mainly within Marine Group II, although between <1 and 12% of reads, depending on the sample, were classified as Marine Group III. Overall, no clear differences were observed in the archaeal lineages detected within the different stations and sampling periods in the nearby of the volcano. The larger differences were observed between epi- and mesopelagic samples. The archaeal groups that typically dominate hydrothermal systems (i.e. Nanoarchaea, Archeaoglobales, Thermococcales, Thermoplasmatales) were not detected in our sequences; however, we cannot discount their presence since we did not perform deep sequencing of Archaea and they may have been present at very low abundances. Unlike the bacterial groups associated with eruptive processes like the *Epsilonproteobacteria* that can grow at mesophilic temperatures, most archaeal groups from hydrothermal systems are from thermophilic to hyperthermophilic [44–45]. We hypothesize that if they had been released with the vent material, they could likely not develop in the water column.

## Conclusions

Monitoring of the waters surrounding the volcano revealed that the eruption promoted an increase in the abundance and activity of prokaryotes, probably as a consequence of warming of the water column and the dramatic changes in seawater chemistry, including an increase in Fe(II) and other nutrients, near the volcano [12–13]. The birth of the volcano resulted also in a decrease in bacterial diversity and in minor changes in bacterioplankton composition but, in contrast, no effects were detected in the archaeal community. Nonetheless, the changes produced by the eruption were temporal and all the microbial parameters analyzed indicate that between January and February the microbial community returned to normal levels. The monitoring of this eruption from the initial unrest represents a unique natural ecosystem scale experiment, which allowed us to determine for the first time the effects of volcanic eruptions on planktonic microbial abundance, activity and diversity.

## Supporting Information

S1 Table Sampling period, geographic location of stations (latitude, longitude) and sampling depths (in m) of samples used for the flow-cytometric analyses included in this study from Bimbache (BBC) and Guayota (GYT) cruises. For comparative analyses, samples were grouped in three categories depending on the location: stations in the control zone (Control), stations in the vicinity of the volcano (Volcano) and stations in other affected areas (Affected). Stations indicated with letter R represent those sampled over several cruises.
(PDF)

S2 Table Average relative abundance (percentage) of different taxa in each of the three types of samples clustered together in the nMDS plot (see Fig. 5): eruption, post-eruption and deep samples. (PDF)

S1 Fig Temperature and oxygen profiles from Station 3R (Volcano) and Station 1R (Control) throughout the Bimbache cruises (BBC3, 4–9 Nov 2011; BBC5, 16–20 Nov 2011; BBC8, 13–15 Jan 2012; BBC10, 9–12 Feb 2012; BBC12, 24–26 Feb 2012). (PDF)

S2 Fig Archaea richness estimates (Chao1) by groups of samples: epipelagic samples from eruption (eruption), epipelagic samples from post-eruption (post-eruption) and mesopelagic samples (deep). (PDF)

S3 Fig Rarefaction analyses of the bacterial 16R rRNA gene sequences clustered at 97% similarity. Operational taxonomic units represented by one tag only (singletons) were discarded from the dataset to avoid potential artifacts in diversity estimates. BBC: Bimbache and GYT: Guayota cruises. St. Station. See Fig. 1 and S1 Table for sample information.
(PDF)

S4 Fig Non-metrical multidimensional (nMDS) analysis based on the OTU distribution of the archaeal dataset. The position of samples reflects how different archaeal assemblages are from each other based on their distance in a two-dimensional plot. Distance is derived from Bray-Curtis similarity coefficients calculated from the square root transformed relative abundance of each OTU.
(PDF)
