# Supplementary material for: Transient Changes in Bacterioplankton Communities Induced by the Submarine Volcanic Eruption of El Hierro (Canary Islands)
Source: PLoS One. 2015 Feb 11;10(2):e0118136. doi: 10.1371/journal.pone.0118136 (PMC4324844; doi:10.1371/journal.pone.0118136)
Supplement: S1 Table — For comparative analyses, samples were grouped in three categories depending on the location: stations in the control zone (Control), stations in the vicinity of the volcano (Volcano) and stations in other affected areas (Affected). Stations indicated with letter R represent those sampled over several cruises. (PDF) [file pone.0118136.s001.pdf]

Table S1. Sampling period, geographic location of stations (latitude, longitude) and sampling depths (in m) of samples used for the flow-cytometric analyses included in this study from Bimbache (BBC) and Guayota (GYT) cruises. For comparative analyses, samples were grouped in three categories depending on the location: stations in the control zone (Control), stations in the vicinity of the volcano (Volcano) and stations in other affected areas (Affected). Stations indicated with letter R represent those sampled over several cruises.

Period	LSTg_Station	Latitude	Longitude	Depths (m)	Category
**Eruption**					
4-9 Nov 2011	BBC3_ST01R	27.65502	-17.91484	5, 75, 900, 400, 1926	Control
	BBC3_ST03R	27.61808	-17.99314	0	Volcano
	BBC3_ST04R	27.62924	-18.00648	5, 25, 50, 75, 150	Volcano
	BBC3_ST05R	27.65880	-18.02904	5, 25, 50, 75, 100, 125	Affected
	BBC3_ST06	27.65528	-18.06668	5, 25, 75	Affected
	BBC3_ST08	27.73048	-18.21604	5, 25, 50, 75	Affected
	BBC3_ST10	27.65512	-18.14060	5, 25, 50, 65, 150	Affected
	BBC3_ST11	27.62102	-18.14046	5, 25, 63, 76, 150	Affected
	BBC3_ST12	27.58118	-18.06668	5, 25, 70	Affected
	BBC3_ST14	27.54400	-17.99032	5, 25, 50, 150, 400, 900	Affected
	BBC3_ST15	27.58110	-17.98878	5, 25, 50, 64, 150, 400, 758	Affected
	BBC3_ST17	27.54330	-18.06252	5, 25, 50, 70, 150, 900	Affected
	BBC3_ST18	27.65512	-18.21464	5, 25, 50, 83, 105, 400, 900	Affected
	BBC3_ST20	27.54616	-18.14120	5, 25, 50, 78, 167	Affected
	BBC3_ST22R	27.62546	-17.98922	20, 75, 90	Volcano
	BBC3_ST23R	27.62476	-17.99452	10, 75, 90, 100	Volcano
	BBC3_ST24R	27.62934	-18.00766	5, 25, 50, 75, 250	Volcano
	BBC3_ST914	27.05712	-18.48694	5, 25, 50, 75, 470, 870	Affected
16-20 Nov 2011	BBC5_ST01	27.75956	-18.18516	5, 25, 50, 75, 150, 400	Affected
	BBC5_ST02	27.77882	-18.22348	5, 25, 58, 75, 100	Affected
	BBC5_ST02	27.77882	-18.22348	5, 25, 58, 75, 100	Affected
	BBC5_ST03	27.82024	-18.20372	5, 25, 50, 75, 100	Affected
	BBC5_ST03R	27.61804	-17.99532	5, 25, 50, 100, 125, 160, 305	Volcano
	BBC5_ST04	27.79504	-18.17132	5, 25, 50, 75, 100	Affected
	BBC5_ST04R	27.62832	-18.00642	5, 25, 50, 75, 125	Volcano
	BBC5_ST05	27.77308	-18.10917	5, 25, 55, 75, 100	Affected
	BBC5_ST05R	27.65920	-18.03010	5, 25, 50, 75, 100, 125, 509	Affected
	BBC5_ST06	27.79882	-18.12584	5, 25, 50, 75, 100	Affected
	BBC5_ST07	27.82291	-18.16480	5, 25, 50, 88, 105	Affected
	BBC5_ST08	27.82448	-18.11716	5, 25, 50, 80	Affected
	BBC5_ST09	27.79866	-18.09206	5, 25, 50, 75, 100	Affected
	BBC5_ST10	27.78872	-18.06280	5, 25, 50, 75	Affected
	BBC5_ST11	27.77924	-18.03965	5, 25, 50, 75, 100	Affected
	BBC5_ST12	27.82092	-18.04384	5, 25, 50, 75, 100	Affected
	BBC5_ST13	27.82720	-18.07732	5, 25, 50, 75, 100	Affected
	BBC5_ST14	27.85314	-18.09638	5, 25, 50, 75, 100	Affected
	BBC5_ST15	27.88246	-18.03948	5, 25, 50, 75, 100	Affected

	BBC5_ST16	27.85118	-18.03480	5, 25, 50, 75, 100	Affected
	BBC5_ST17	27.82640	-18.01242	5, 25, 50, 75, 100	Affected
	BBC5_ST18	27.85000	-17.98460	5, 25, 50, 75, 100	Affected
	BBC5_ST19	27.87785	-17.99131	5, 25, 50, 73, 100	Affected
**Posteruption**					
13-15 Jan 2012	BBC8_ST01	27.76958	-17.89514	5, 44, 75, 100, 150, 300	Affected
	BBC8_ST01R	27.65582	-17.91504	5, 50, 75, 100, 150, 300	Control
	BBC8_ST02	27.73426	-17.91492	5, 25, 75, 100, 150, 300	Affected
	BBC8_ST02R	27.61848	-17.91458	5, 25, 75, 100, 150, 300	Affected
	BBC8_ST03	27.65600	-17.95796	5, 25, 75, 100, 150, 300	Affected
	BBC8_ST03R	27.61835	-17.98932	5, 25, 50, 100	Volcano
	BBC8_ST04	27.61924	-17.95676	5, 50, 75, 100, 150	Affected
	BBC8_ST04R	27.62860	-18.00580	5, 50, 75, 150, 310	Volcano
	BBC8_ST05	27.68310	-17.95870	5, 50, 75, 100, 150, 200, 300	Affected
	BBC8_ST05R	27.65910	-18.02830	5, 50, 75, 86, 150, 300	Affected
	BBC8_ST06	27.67977	-17.91742	5, 50, 75, 100, 150, 300	Affected
	BBC8_ST07	27.70700	-17.91792	5, 50, 85, 100, 150, 300	Affected
	BBC8_ST08	27.70596	-17.94732	5, 50, 75, 100, 150, 300	Affected
	BBC8_ST09	27.74858	-17.88124	5, 50, 75, 100, 150, 300	Affected
	BBC8_ST21R	27.61062	-17.99748	5, 50, 75, 100, 150, 300	Volcano
	BBC8_ST23R	27.62478	-17.99454	5, 57, 75, 100, 150	Volcano
9-12 Feb 2012	BBC10_ST01	27.68432	-18.06760	5, 25, 50, 75, 100, 150, 200, 400, 800	Affected
	BBC10_ST02	27.65778	-18.06673	5, 50, 150, 200, 400, 500	Affected
	BBC10_ST03	27.62938	-18.06676	5, 25, 50, 75, 100, 150, 200, 400, 500	Affected
	BBC10_ST03R	27.62008	-17.99550	5, 25, 50, 75, 100, 125, 150, 160	Volcano
	BBC10_ST04	27.61194	-18.06758	5, 50, 150, 200, 400, 500	Affected
	BBC10_ST04R	27.62844	-18.00556	5, 50, 75, 100, 150, 200	Volcano
	BBC10_ST05	27.59048	-18.06532	5, 50, 150, 200, 400, 500	Affected
	BBC10_ST05R	27.65796	-18.02892	5, 25, 50, 75, 100, 150	Affected
	BBC10_ST06	27.62838	-18.02784	5, 50, 165, 200, 400, 500	Affected
	BBC10_ST07	27.60978	-18.02658	25, 5, 100, 200, 400, 500	Affected
	BBC10_ST08	27.58518	-18.02578	5, 56, 150, 200, 400, 500	Affected
	BBC10_ST21R	27.61130	-17.99658	5, 25, 50, 75, 100, 150	Affected
	BBC10_ST23R	27.62516	-17.99524	5, 150, 215	Volcano
24-26 Feb 2012	BBC12_ST01	27.62830	-18.02660	5, 50, 100, 200, 300, 400	Affected
	BBC12_ST01R	27.65724	-17.91360	5, 75, 125, 400, 600, 800	Control
	BBC12_ST02	27.60914	-18.02592	5, 50, 100, 200, 300, 400	Affected
	BBC12_ST03	27.58776	-17.99796	5, 50, 100, 200, 300, 400	Affected
	BBC12_ST03R	27.61988	-17.99266	5, 20, 25, 30, 50, 70, 75, 86, 100, 165, 185	Volcano
	BBC12_ST04	27.58530	-18.02660	5, 50, 100, 200, 300, 400	Affected
	BBC12_ST04R	27.62880	-18.00540	5, 25, 40, 100, 200, 300	Volcano
	BBC12_ST05	27.61137	-18.00320	5, 25, 50, 75, 100, 127	Affected
	BBC12_ST05R	27.65883	-18.02874	5, 25, 50, 100, 200, 300	Affected
	BBC12_ST06	27.62010	-18.00296	5, 50, 100, 200, 300, 423	Affected

	BBC12_ST07	27.62082	-17.98715	5, 25, 50, 75, 100, 150	Affected
	BBC12_ST08	27.63324	-17.98424	5, 25, 38	Affected
	BBC12_ST21R	27.61020	-17.99784	5, 50, 100, 200, 300, 400	Volcano
17 Mar 2012	GYT2_ST04R	27.62850	-18.00567	5, 25, 60, 100, 125, 250	Volcano
	GYT2_ST23R	27.62467	-17.99467	5, 35, 75, 100	Volcano
	GYT3_ST03R	27.61833	-17.98917	5, 25, 50, 75, 100, 115, 150, 250	Volcano
28 Apr 2012	GYT3_ST21R	27.61050	-17.99733	5, 50, 75, 100, 200, 300	Volcano
	GYT3_ST23R	27.62467	-17.99467	5, 50, 75, 100, 150, 180	Volcano
