# Supplementary material for: Transient Changes in Bacterioplankton Communities Induced by the Submarine Volcanic Eruption of El Hierro (Canary Islands)
Source: PLoS One. 2015 Feb 11;10(2):e0118136. doi: 10.1371/journal.pone.0118136 (PMC4324844; doi:10.1371/journal.pone.0118136)
Supplement: S2 Table — (PDF) [file pone.0118136.s002.pdf]

Table S2. Average relative abundance (percentage) of different taxa in each of the three types of samples clustered together in the nMDS plot (see Figure 5): eruption, post-eruption and deep samples.

**TAXA**	ERUPTION	POST-ERUPTION	DEEP
Actinobacteria	1.1	2.3	2.8
Bacteroidetes	3.9	7.1	1.8
Candidate division ZB3	0.2	0.0	0.0
Chloroflexi (SAR202 group)	0.2	0.2	2.7
Cyanobacteria	35.7	13.2	0.3
Gemmatimonadetes	0.0	0.0	0.4
Planctomycetes	0.2	0.1	0.5
Proteobacteria: Alphaproteobacteria-SAR11 group	39.2	56.0	48.2
Proteobacteria: Alphaproteobacteria-Other	2.8	2.7	3.2
Proteobacteria: Betaproteobacteria	0.2	0.1	0.0
Proteobacteria: Deltaproteobacteria	0.7	0.5	17.4
Proteobacteria: Epsilonproteobacteria	1.5	0.0	0.2
Proteobacteria: Gammaproteobacteria	11.0	10.2	7.6
SAR406	1.4	1.6	6.5
Verrucomicrobia	0.8	0.3	0.5
Bacteria: Other*	1.1	5.7	7.9

*Includes: Zetaproteobacteria, Spirochaetes, Firmicutes, Acidobacteria, Nitrospirae, Lentisphaerae and other unclassified Bacteria
