# Supplementary material for: Transient Changes in Bacterioplankton Communities Induced by the Submarine Volcanic Eruption of El Hierro (Canary Islands)
Source: PLoS One. 2015 Feb 11;10(2):e0118136. doi: 10.1371/journal.pone.0118136 (PMC4324844; doi:10.1371/journal.pone.0118136)

Figure S1. Temperature and oxygen profiles from Station 3R (Volcano) and Station 1R (Control) throughout the Bimbache cruises (BBC3, 4-9 Nov 2011; BBC5, 16-20 Nov 2011; BBC8, 13-15 Jan 2012; BBC10, 9-12 Feb 2012; BBC12, 24-26 Feb 2012).

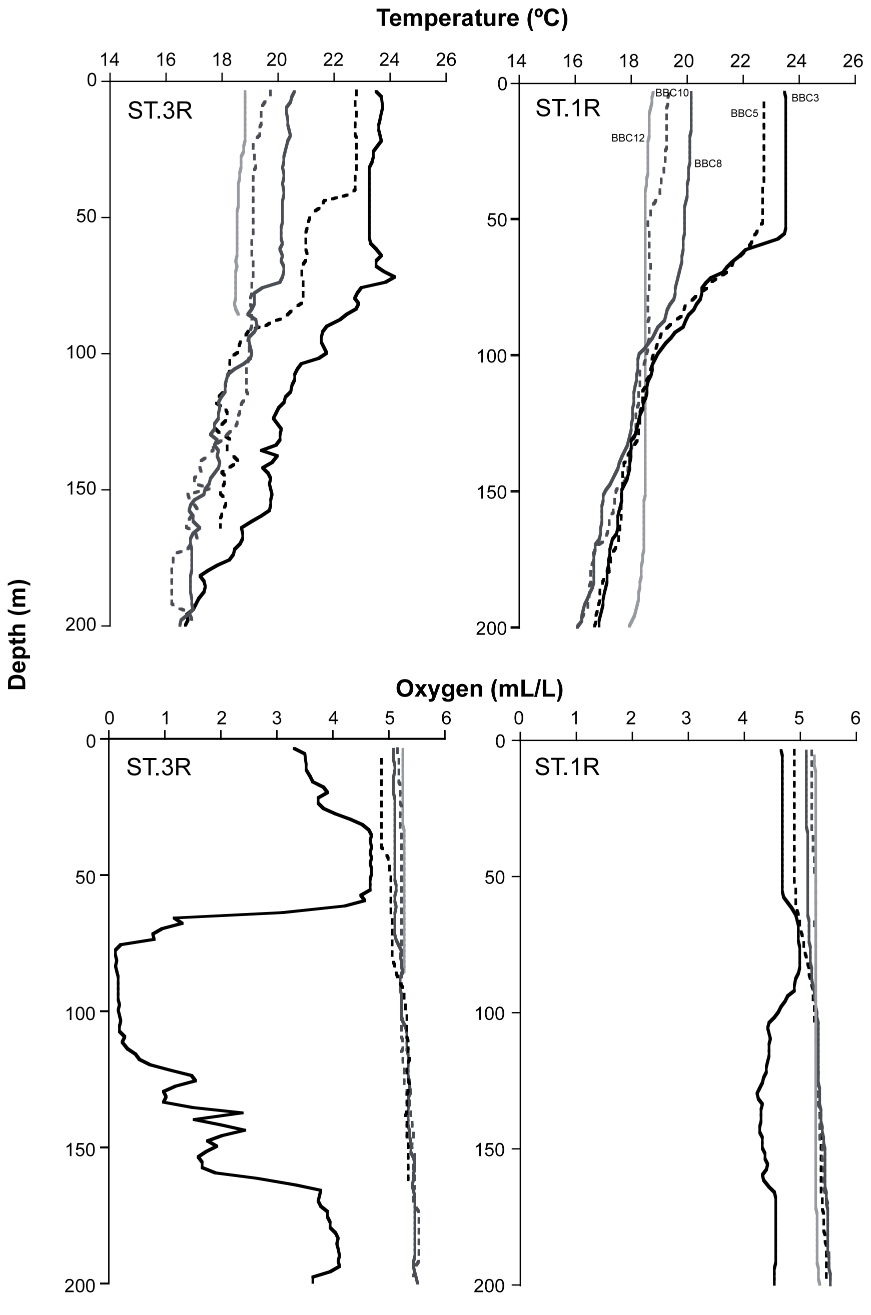

Supplement: S1 Fig — (PDF) [file pone.0118136.s003.pdf]
