# Supplementary material for: Transient Changes in Bacterioplankton Communities Induced by the Submarine Volcanic Eruption of El Hierro (Canary Islands)
Source: PLoS One. 2015 Feb 11;10(2):e0118136. doi: 10.1371/journal.pone.0118136 (PMC4324844; doi:10.1371/journal.pone.0118136)

Figure S2. Archaea richness estimates (Chao1) by groups of samples: epipelagic samples from eruption (eruption), epipelagic samples from post-eruption (post-eruption) and mesopelagic samples (deep).

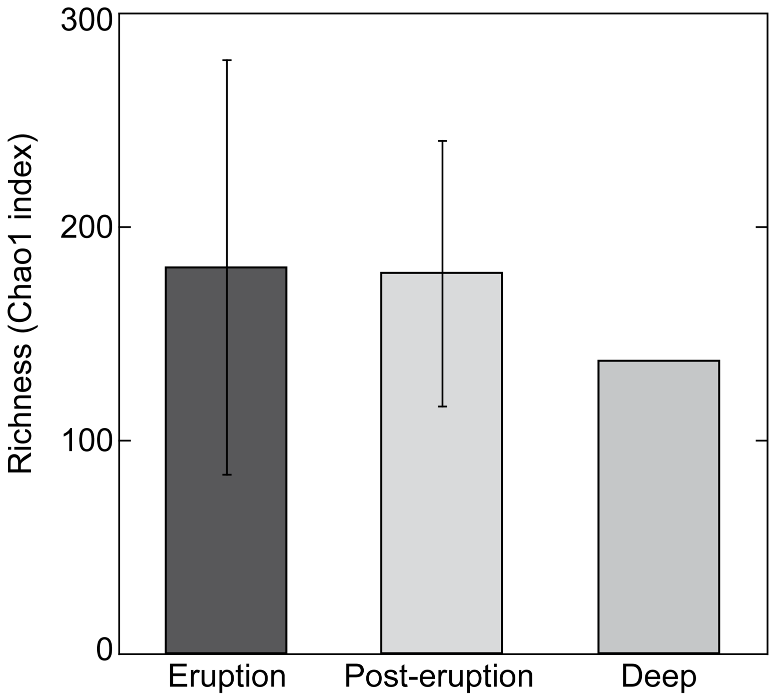

Supplement: S2 Fig — (PDF) [file pone.0118136.s004.pdf]
