# Supplementary material for: Transient Changes in Bacterioplankton Communities Induced by the Submarine Volcanic Eruption of El Hierro (Canary Islands)
Source: PLoS One. 2015 Feb 11;10(2):e0118136. doi: 10.1371/journal.pone.0118136 (PMC4324844; doi:10.1371/journal.pone.0118136)

Figure S3. Rarefaction analyses of the bacterial 16R rRNA gene sequences clustered at 97% similarity. Operational taxonomic units represented by one tag only (singletons) were discarded from the dataset to avoid potential artifacts in diversity estimates. BBC: Bimbache and GYT: Guayota cruises. St. Station. See Figure 1 and Supplementary Table SM1 for sample information.

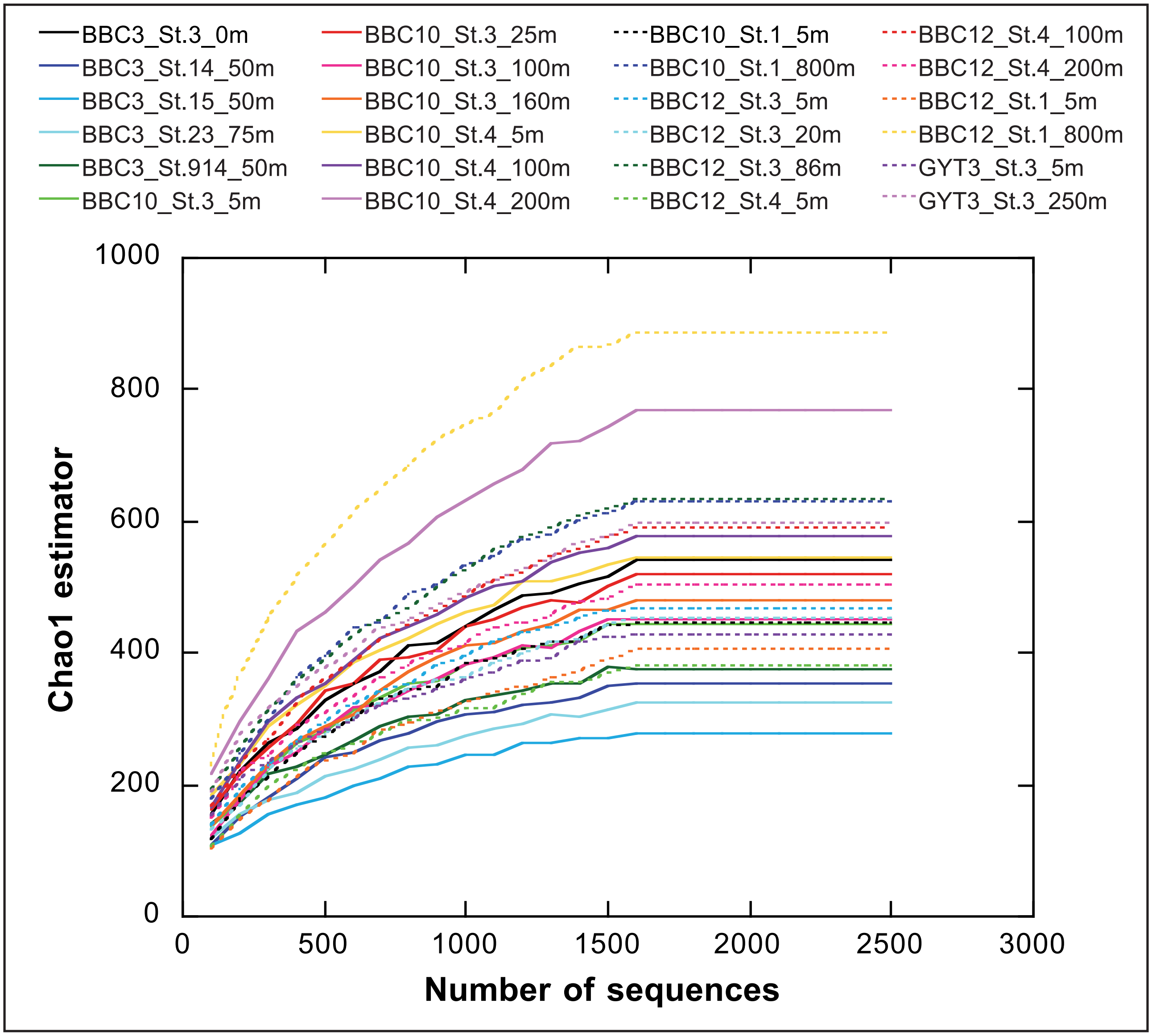

Supplement: S3 Fig — Operational taxonomic units represented by one tag only (singletons) were discarded from the dataset to avoid potential artifacts in diversity estimates. BBC: Bimbache and GYT: Guayota cruises. St. Station. See Fig. 1 and S1 Table for sample information. (PDF) [file pone.0118136.s005.pdf]
