# Supplementary material for: Transient Changes in Bacterioplankton Communities Induced by the Submarine Volcanic Eruption of El Hierro (Canary Islands)
Source: PLoS One. 2015 Feb 11;10(2):e0118136. doi: 10.1371/journal.pone.0118136 (PMC4324844; doi:10.1371/journal.pone.0118136)

Figure S4. Non-metrical multidimensional (nMDS) analysis based on the OTU distribution of the archaeal dataset. The position of samples reflects how different archaeal assemblages are from each other based on their distance in a two-dimensional plot. Distance is derived from Bray–Curtis similarity coefficients calculated from the square root transformed relative abundance of each OTU.

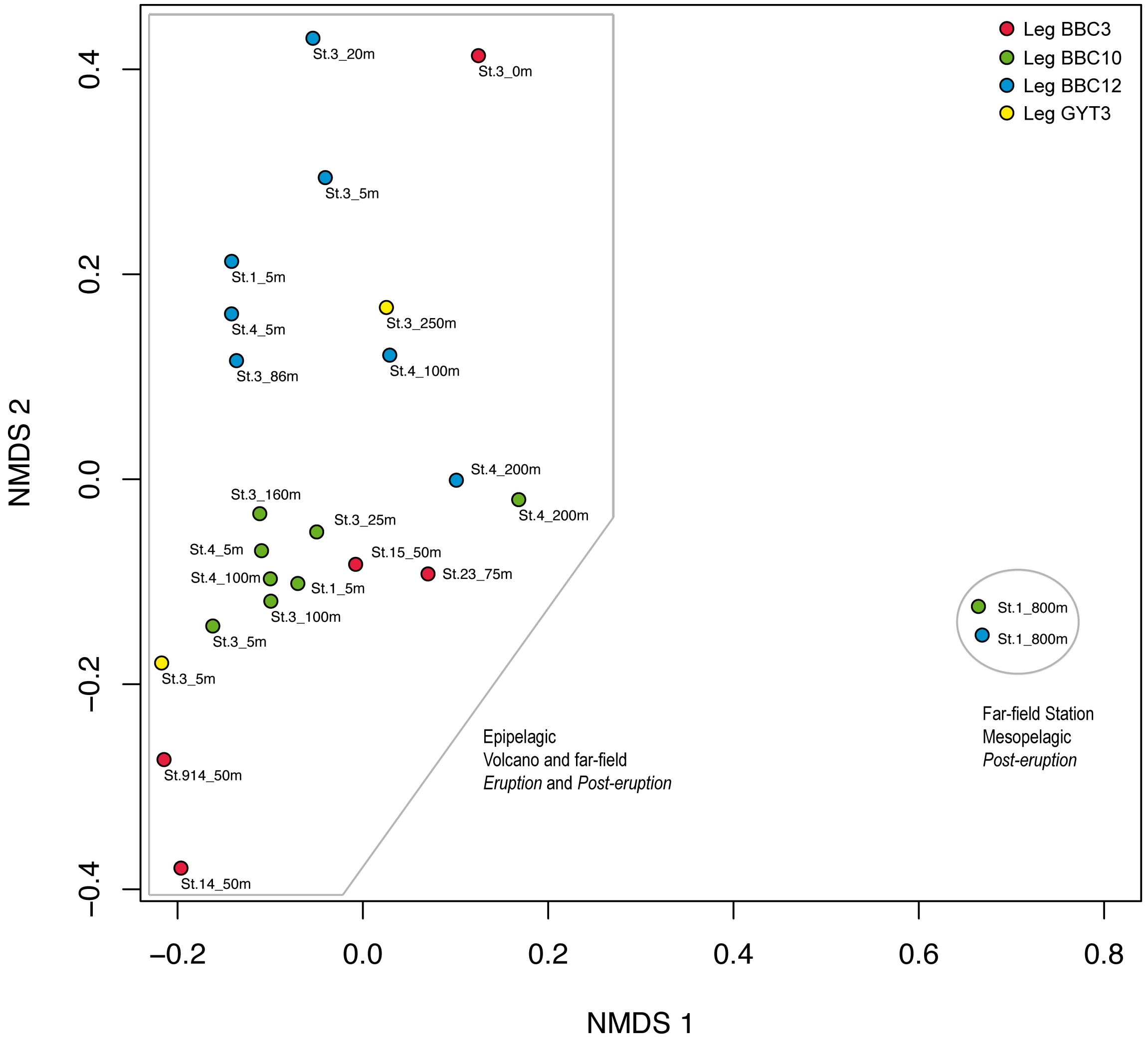

Supplement: S4 Fig — The position of samples reflects how different archaeal assemblages are from each other based on their distance in a two-dimensional plot. Distance is derived from Bray-Curtis similarity coefficients calculated from the square root transformed relative abundance of each OTU. (PDF) [file pone.0118136.s006.pdf]
